# Periprostatic Adipose Tissue as a Contributor to Prostate Cancer Pathogenesis: A Narrative Review

**DOI:** 10.3390/cancers17030372

**Published:** 2025-01-23

**Authors:** Julia Drewa, Katarzyna Lazar-Juszczak, Jan Adamowicz, Kajetan Juszczak

**Affiliations:** 1 Department of Urology and Andrology, Collegium Medicum, Nicolaus Copernicus University, 85-094 Bydgoszcz, Poland; 2 Primary Health Care Clinic of the Ujastek Medical Center, 31-752 Cracow, Poland; 3 Krakow University of Health Promotion, 31-158 Cracow, Poland; 4 Department of Regenerative Medicine, Collegium Medicum, Nicolaus Copernicus University, 85-094 Bydgoszcz, Poland

**Keywords:** periprostatic adipose tissue, prostate cancer, obesity, pathogenesis, adipokines

## Abstract

Periprostatic adipose tissue (PPAT) contributes to the pathogenesis of prostate cancer. The purpose of this study was to review and summarize the literature on the role of PPAT in prostate cancer pathogenesis. Moreover, we evaluated the clinical implication of PPAT in patients with prostate cancer. We performed a scoping literature review of PubMed from January 2002 to November 2024. Search terms included “periprostatic adipose tissue”, “adipokines”, and “prostate cancer”. Secondary search involved reference lists of eligible articles. The key criterion was to identify studies that included PPAT, adipokines, and their role in prostate cancer biology and clinical features. In total 225 publications were selected for inclusion in this review. The studies contained in publications allowed us to summarize the data on the pathogenesis of PPAT as a contributor to prostate cancer biology and its aggressiveness. The review also presents new research directions for PPAT as a new target for the treatment of prostate cancer. Based on the current review, it can be stated that PPAT plays an important role in prostate cancer pathogenesis. Moreover, PPAT seems to be a promising target point when it comes to finding new therapies in patients with more aggressive and/or advanced stages of prostate cancer.

## Simple Summary

The role of periprostatic adipose tissue in the pathogenesis of prostate cancer seems to be unquestionable. Moreover, periprostatic adipose tissue is involved in the progression of prostate cancer. Our literature review provides detailed information on the involvement of periprostatic adipose tissue in the development of prostate cancer. The data so far indicate that periprostatic adipose tissue may become an attractive target for modern prostate cancer therapies, especially in patients with more aggressive and/or advanced stages of prostate cancer. Nevertheless, further research is necessary in this field.

## 1. Introduction

Prostate cancer is the second most commonly diagnosed cancer in men [1]. In the European male population, prostate cancer remains the most frequently diagnosed cancer (excluding skin cancer) [2]. Prostate cancer is diagnosed on the basis of a prostate biopsy. Laboratory tests (assessment of the PSA level) and imaging tests (multiparametric magnetic resonance imaging) are helpful in diagnosis. Urine and serum biomarkers as well as tissue-based biomarkers have been proposed for improving detection and risk stratification of prostate cancer patients, potentially avoiding unnecessary biopsies. Most patients with prostate cancer require radical treatment (radical prostatectomy). However, taking into account the biology of prostate cancer and the clinical features of patients with prostate cancer (age, comorbidities, stage of the disease, etc.) in selected cases, we choose non-surgical methods (e.g., radical radiotherapy, hormonotherapy, chemotherapy, etc.). The pathogenesis of prostate cancer is multi-factorial but still incompletely understood [3]. Several etiological factors have been discussed as being associated with the risk of developing prostate cancer or as being etiologically important for the progression from latent to clinical prostate cancer [4]. The possible modifiable risk factors for the development of prostate cancer include metabolic syndrome, obesity, diet, and smoking [5].

A comprehensive meta-analysis provides epidemiological evidence supporting the association between body mass index (BMI) and cancer risk and reports an increased risk of aggressive prostate cancer in obese patients [6]. Despite advances in prostate cancer diagnosis and management, morbidity from prostate cancer still remains high. Despite hormonal therapy, patient with prostate cancer progress to castration-resistant prostate cancer (CRPC) approximately within 2–3 years of initiation of androgen deprivation therapy [7]. Bone metastases will occur in about 90% of patients with CRPC [8]. Therefore, there is a need for new therapeutic targets in patients witch prostate cancer, especially in CRPC clinical stage. Periprostatic adipose tissue (PPAT) contributes to the pathogenesis of prostate cancer development. Moreover, it is postulated that PPAT is involved in prostate cancer progression into metastatic disease.

The following review aims to discuss and provide information about the role of PPAT in prostate cancer pathogenesis and its clinical implication in patients with prostate cancer. The studies contained in publications allowed us to summarize the data on the pathogenesis of PPAT as a contributor to prostate cancer biology and its aggressiveness. The review also presents new research directions for PPAT as a new target for the treatment of prostate cancer, especially in patients with more aggressive and/or advanced stages of prostate cancer.

## 2. Obesity and Cancer

Obesity is strictly related to several chronic diseases, especially cardio-vascular illnesses, diabetes, sleep apnea, and osteoarthritis [9]. It is worth noting that there is growing evidence that overweight and obesity is a potential risk factor for cancer development [10]. Obesity attributes to about 4–8% of all cancers [11]. Previous studies showed the link between obesity and gastro-intestinal cancers (e.g., esophagus, stomach, colon, and rectum), gallbladder and pancreas cancer, genito-urinary cancers (e.g., prostate, uterus, and ovary), and breast cancer [11,12,13,14]. Aune et al.’s [15] study revealed that overweight and obesity is associated with increased risk of all-cause mortality. Additionally, obesity was associated with greater risk of recurrence and mortality overall in patients with cancer [16,17]. 

The potential pathomechanisms of obesity causing cancer are complex and multifactorial. This phenomenon remains incompletely explained. Obesity results from a chronic excessive accumulation of adipose tissue. In the literature, several potential cellular and molecular pathomechanisms linking cancer development, as well as cancer progression and metastasis development, to obesity were described [18]. These mechanisms include, among others, micro-environmental disturbances and extracellular matrix remodeling, altered fatty acid metabolism, hormonal disturbances (e.g., anabolic hormone), chronic inflammation, and immune instability [19]. Additionally, Renehan et al. [20] proposed several hormonal pathomechanisms attributed to cancer development, as follow: (1) adipokine-related pathways, (2) altered sex hormone metabolism, and (3) elevated insulin level and increased bioavailability of insulin-like growth factor 1 (IGF-1). Moreover, it is postulated that interstitial microbiota dysregulation is important in obesity, obesity-related diseases, and cancer development [21]. Roger et al. [22] draws attention to intestinal microbiome dysregulation affect proper bacterial metabolism, which may lead to production of potential procarcinogenic metabolites. Additionally, metabolic dysfunction due to boost energy extraction, nutrient availability, and induction of inflammation seems to contribute to tumor cells development and/or growth.

## 3. Obesity and Prostate Cancer

The relationship between obesity and the risk of prostate cancer is still unclear. Previous studies confirmed that obese men are at higher risk for dying of prostate cancer [23]. Moreover, men with obesity predispose to more aggressive form of prostate cancer [24,25]. Previous meta-analyses indicate a positive association between prostate cancer and obesity. In general, the relative risks (RRs) were from 1.01 (95% confidence interval [CI], 1.0–1.02) per 1 kg/m^2^ increase in BMI [26] to 1.05 (95% CI, 1.01–1.08) [25] and 1.03 (95% CI, 1.0–1.07) [27] per 5 kg/m^2^ increment in BMI [24]. 

There are two types of adipose tissue: white adipose tissue (WAT) and brown adipose tissue (BAT). Moreover, third type of adipose tissue can be distinguished: a “beige” adipose tissue due to the “browning” of WAT phenomenon. Many studies indicate the active participation of adipose tissue in the development and progression of prostate cancer via endocrine and paracrine signalling [28]. Hond et al. [29] study showed that that adipose tissue covered about 48% of the prostate gland surfaces.

Allott et al. [24] study described a close positive correlation among prostate cancer and other comorbidities (e.g., metabolic syndrome and obesity). There are currently several potential mechanisms explaining the relationship between obesity and the development of prostate cancer. First, generalized and local inflammatory response stimulated by immune system cells present in the adipose tissue, including that surrounding the prostate gland. Second, obesity and excess adipose tissue lead to the development of hyperinsulinemia. Third, excess adipose tissue predisposes to changes in the availability of a number of substances with para- and endocrine effects, including the so-called adipokines [30]. White adipose tissue surrounding the prostate gland forms the so-called PPAT and is one of the most important components of the prostate microenvironment, taking an active part in the physiological conditions as well as in the development of prostate cancer. Namely, the periprostatic adipose tissue is an active endocrine tissue, influencing the activity of prostate cells, but above all, by modulating the microenvironment, it promotes the processes of migration, invasion, and aggressiveness of prostate cancer cells [30,31].

In people with normal body weight, a proper balance between pro- and anti-inflammatory pathways is maintained in the adipose tissue. Thanks to the secretion of greater amounts of anti-inflammatory substances from the extracellular matrix and the adipose cells themselves [32]. As body weight increases, a predominance of pro-inflammatory activity is observed. This is due to increased production of adipokines associated with developing insulin resistance and increased inflammation. In addition, the cell death of some hypertrophic adipocytes and the release of intracellular substances are observed, which increase the already existing inflammation, intensifying the pro-inflammatory response [33]. 

Early childhood and puberty are important periods for the development of normal adipose tissue mass. After puberty, adipocyte number and size become static in lean individuals [34]. In obese subjects (in development and adulthood), the increment in adipose tissue mass is due to adipocyte size (hypertrophy) and increased adipocyte number (hyperplasia) [35]. In adults, a high-fat diet transiently induces adipocyte precursors to proliferate, which leads to the increased formation of adipocytes in diet-induced obesity. The development of PPAT appears to be parallel with the development of obesity, particularly of the visceral type. The increase in PPAT mass results mainly from the enlargement of adipocytes already present there. However, the coexistence of adipocyte proliferation cannot be ruled out. The extent of PPAT seems to be related to the degree of visceral obesity due to their morphological and functional similarities. Obesity is associated with changes in adipocyte gene expression spanning many pathways. These changes alter fuel partitioning between adipose tissue and other tissues. In addition, they alter the hormonal milieu by changing the relative expression of adipose hormones, adipokines. Finally, many metabolites act as signaling molecules; thus, their redistribution alters signaling pathways in adipose and other tissues [36].

## 4. PPAT and Prostate Cancer Biology

Adipocytes contained in PPAT are thought to influence cancer cell activity via pathological feedback loops dependent on paracrine secretion of adipokines. These adipokines influence cancer cell growth and survival. Park et al. [30] described the close interactions of cancer cell and adipocytes via endocrine and paracrine pathways. Several proposed pathophysiological mechanisms of prostate cancer tumorigenesis have been described at the cellular level, including: (1) the insulin and IGF axis, (2) deregulated adipokine signalling, and (3) the expansion of adipose/stromal stem cells population [37,38,39]. It is worth remembering, however, that the “adipocytes—prostate cancer cells” interaction is not one-way. Several studies have confirmed the observation that prostate cancer cells demonstrate the ability to induce genetic and phenotypic changes in neighbouring adipocytes, leading to their transformation into so-called “cancer-associated adipocytes” [40]. During tumour growth, epithelial cells leave the glandular niche and invade the microenvironment becoming exposed to huge concentrations of adipokines released by PPAT that boost their proliferation [28,41].

**Figure 1:**
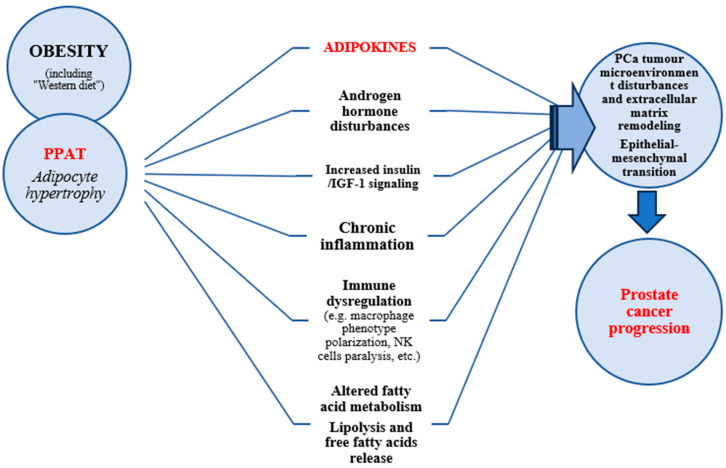
Obesity-associated pathomechanisms of prostate cancer development. PPAT—Periprostatic adipose tissue, IGF-1—Insulin-like growth factor-1, NK—Natural Killer, PCa—prostate cancer.

At the same time, remodelling of the extracellular matrix seems to be an inherent pathophysiological mechanism influencing tumour development and progression. Moreover, obesity predispose to infiltration of adipose tissues by inflammatory cells (e.g., macrophages and leukocytes) and in a consequence trigger the local and systemic inflammatory response and insulin resistance [42]. During local inflammatory process the reactive oxygen species are generated that act as tumour promoters at a low concentration [43]. Adipocytes, stromal cells, and infiltrating inflammatory cells in adipose tissue secrete several adipokines and other cytokines, which play a crucial role in the development of obesity-related cancer [42,44] (Figure 1).

## 5. PPAT and Adipokines 

**Figure 2:**
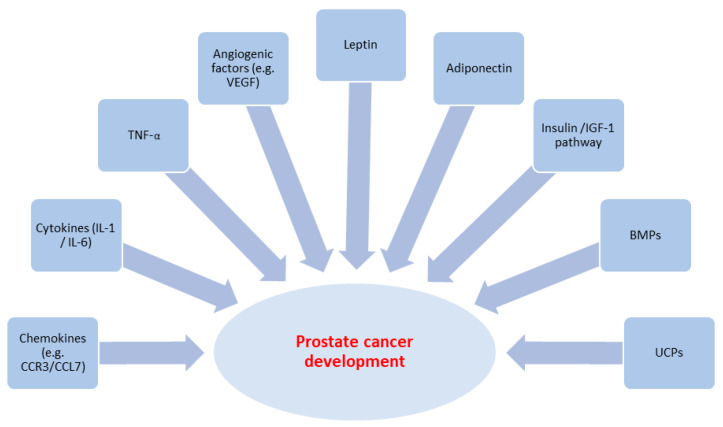
Adipokines released from PPAT affect prostate cancer development. CCR3—chemokine receptor 3, CCL7—chemokine ligand 7, IL-1—interleukin-1, IL-6—interleukin-6, TNF-α—tumor necrosis factor α, VEGF—vascular endothelial growth factor, IGF-1—Insulin-like growth factor-1, BMPs—Bone Morphogenetic Proteins, UCPs—mitochondrial uncoupling protein.

Adipose tissue releases a number of substances named adipokines. The adipokines include chemokines, cytokines (e.g., interleukin-1 (IL-1), interleukin-6 (IL-6) and tumour necrosis factor (TNF)-α), angiogenic factors (e.g., vascular endothelial growth factor (VEGF), and other substances (e.g., leptin, adiponectin, and insulin-like growth factor 1 (IGF1), etc.) [28,42] (Figure 2).

### 5.1. Chemokines

Chemokines are small molecules that function as chemotactic factors, which regulate the migration, infiltration, and accumulation of immune cells [45]. Laurent et al.’s [46] study showed that PPAT, especially its adipocytes, is directly involved in prostate cancer cell migration outside the prostate gland. Additionally, obesity promotes this migration. There are several adipokines involved in prostate cancer cell migration. The chemokine CCL7 secreted by adipocytes is pivotal in extensive migration. CCL7 diffuses through the prostatic capsule to the peripheral zone of the prostate gland and consequently stimulates the migration of the CCR3 = positive prostate cancer cells outside the prostate. Obesity predisposes to higher secretion of chemokines (e.g., CCL7) by hypertrophic adipocyte cells from PPAT and secondarily facilitates extraprostatic extension, leading to locally advanced disease. Additionally, CCR3/CCL7 axis blockade leads to inhibition of increased obesity-related cancer cell migration. The expression of the CCR3 receptor is associated with the occurrence of aggressive disease with extended local dissemination and a higher risk of biochemical recurrence of prostate cancer. 

The adipocytes release the stromal cell derived factor 12 (CXCL12) through direct interaction with the chemokine receptor CXC type 4 (CXCR4) present in the membrane of tumour cells activates the intracellular cascade of gene transcription involved in cell survival, or migration and invasion, thus promoting EMT [28]. The process of epithelial–mesenchymal transition (EMT) plays a pivotal role in the development of metastatic castration resistant prostate cancer (m CRPC) [47]. The epithelial–mesenchymal transition (EMT) in important in prostate tumours growth and progression [48]. Moreover, the EMT phenomenon is promoted by obesity [49]. 

### 5.2. Leptin

Leptin is an obesity-associated adipokine. This molecule regulates energy metabolism and reproduction, as well as controls an appetite via the leptin receptor. Moreover, cancer cells and the tumour microenvironment expressing leptin and leptin receptors suggest that the potential leptin autocrine/paracrine signalling loop could affect tumour progression [50]. Gorrab et al. [51] demonstrated that high doses of leptin promote prostate cancer cell migration and EMT transition via stimulation of the STAT3 pathway. Moreover, the blockage of leptin receptors inhibits prostate cancer xenograft growth and progression in a mice model [52]. Cellular invasion and migration of androgen-resistant prostate cancer cells (PC-3 and DU-145 cell lines) are attenuated by leptin in a concentration-dependent manner in prostate cancer cell lines. Additionally, the proliferation of androgen-dependent and independent prostate cancer cell lines was observed in the presence of nanogram concentrations of leptin [53]. Serum levels of leptin positively correlate with prostate cancer development [54]. Chang et al. [55] study revealed that men with high-volume disease exhibited higher serum leptin concentrations overall and after stratification by age, testosterone level, height, and body mass index (BMI).

### 5.3. Adiponectin

Adiponectin interacts via ADIPOR1 and ADIPOR2 membrane receptors [56]. Adiponectin has significant anti-diabetic, anti-inflammatory, anti-atherosclerotic and anti-proliferative properties [54]. Patients with prostate cancer have lower serum adiponectin levels and decreased expression of adiponectin receptors in tumour tissues, which suggests that plasma adiponectin level is a risk factor for prostate cancer. Prostate cancer patients had significantly lower plasma adiponectin concentrations as compared to men with benign prostate hyperplasia and healthy controls. Men in the top two quartiles of adiponectin had a 71% to 73% reduced risk of prostate cancer as compared to men in the lowest quartile [57]. Li et al. [58] study showed that adiponectin concentrations were not associated with overall risk of prostate cancer. However, men with higher adiponectin concentrations had lower risk of developing high-grade or lethal cancer (metastatic or fatal disease). Endogenous adiponectin may function as a tumour suppressor gene through inhibiting the epithelial–mesenchymal transition of prostate cancer cells but is down-regulated in prostate cancer via promoter hypermethylation [59]. Contrarily, Tang et al.’s [60] study showed that adiponectin increased the migration and the expression of α5β1 integrin of human prostate cancer cells. One of the mechanisms underlying directed adiponectin migration was transcriptional up-regulation of α5β1 integrin and activation of AdipoR1 receptor, p38, AMPK, and NF-κB pathways. Previous studies revealed that that adiponectin presents anti-proliferative properties in prostate cancer cells and inhibits dihydrotestosterone-activated cell proliferation, IL-6, and IGF-I [61,62,63].

### 5.4. Insulin-like Growth Factor 1

Insulin-like growth factor (IGF)-1 is associated with a higher risk of prostate cancer [64]. Similarly, Qian et al. [65] revealed that the elevated serum IGF-1 levels increase the future risk of prostate cancer development in healthy men. Acromegaly patients with systemically high growth hormone (GH) and IGF-1 levels also have significantly higher incidence of prostate cancer and risk of prostate cancer-related mortality (HR = 1.33 and 1.44, respectively), suggesting that IGF-1 has a positive effect on prostate cancer development and progression [66]. IGF-1 is inhibited by IGF-binding protein (IGFBP)-1, which is defined as a marker for insulin activity. Insulin is responsible for proper carbohydrate and energy metabolism. It also has a strong mitogenic effect and stimulates prostate growth, which may contribute to the development of prostate cancer [67]. On the one hand, epidemiological studies to date clearly indicate the relationship between higher levels of circulating IGF-1 and an increased risk of prostate cancer development (especially low-grade cancer). On the other hand, the potential pathomechanisms related to insulin and IGF pathways remain unclear [68,69]. In patients with prostate cancer, the overexpression of IGF-1 receptor is observed. IGF-1 accelerated the growth of prostate cancer by activating phosphoinositide 3-kinase and mitogen-activated protein kinase or increasing sex hormone sensitivity [70]. Cao et al.’s [64] results showed that higher fasting IGF-binding protein (IGFBP-1) levels were associated with lower risk of prostate cancer, whereas higher IGF-1 levels were associated with increased prostate cancer risk. The associations were primarily driven by lower-grade and non-advanced prostate cancer. In patients with locally advanced and metastatic prostate cancer, androgen deprivation therapy remains very important therapeutical option. Experimental data confirmed the implication of IGF-1 axis in prostate cancer development. Moreover Ravi et al. [71] evaluate the association between IGF-1 and its binding proteins on outcomes in men with metastatic prostate cancer treated with androgen deprivation therapy, with or without docetaxel. The observations showed that a higher baseline and 6-month IGF-1:IGF-BP1 ratio was associated with better overall survival. 

The normal function of white adipose tissue (WAT) is to store and release lipids. In obesity, WAT develops inflammation, fibrosis, and dysfunction and is sufficient to enhance cancer progression irrespective of diet. Periprostatic WAT, more abundant in obese patients, plays a particularly important role in prostate cancer [72]. Moreover, adipose tissue is considered to be more metabolically active than other tissues and to serve a prominent role in prostate cancer development. Visceral adipocyte tissue produces multiple molecules. PPAT is a type of visceral adipose tissue which serves an important role in prostate cancer biology. The ability of PPAT to secrete a number of molecules (cytokines, hormones, IGF-1, etc.) makes this tissue extremely similar to visceral adipose tissue [73].

In obesity pathogenesis, growth hormone (GH)/insulin-like growth factor (IGF)-dependent pathways remain crucial. Both GH and IGF-I have direct effects on adipocyte proliferation and differentiation. Insulin resistance, which is commonly associated with obesity, has been shown to promote prostate cancer by increasing circulating levels of bioactive IGF-1, a growth factor implicated in numerous types of cancer [74,75]. Yang et al.’s [76] study showed that metformin intake significantly increased IGF-1 level. On the other hand, Tosca et al.’s [77] study revealed that metformin reduces cell growth, protein synthesis, MAPK3/1, and P90RSK phosphorylation in response to IGF1 through an AMPK-dependent mechanism in cultured bovine granulosa cells. However, the influence of metformin on prostate cancer biology remains inconclusive [78]. Kim et al. [79] did not identify a significant positive correlation between metformin and prostate cancer. However, preoperational PSA and PSA velocity tended to be lower in the metformin cohort.

### 5.5. Interleukins

In obese patient the elevated level of IL-1 is observed [28]. It is postulated that IL-1 is a risk factor of cancer (e.g., colorectal and lung cancer) [80]. IL-1 is released by the activated macrophages present in the tumour microenvironment, but it is also produced by adipose tissue [81]. Proinflammatory cytokines can influence neuroendocrine differentiation in prostate cancer and be involved in disease progression [82]. Inflammation is known to promote tumour formation and progression. Fan et al. [83] found a natural anti-inflammatory factor, interleukin (IL)-1 receptor antagonist (IL1RN), in a mouse transgenic adenocarcinoma of the mouse prostate (TRAMP)-C1-derived tumour microenvironment.

Interleukin 6 (IL-6). IL-6 is also released by adipocytes in case of obesity. Interestingly, it has been found that although IL-6 is primarily considered a pro-inflammatory cytokine, it also has many regenerative or anti-inflammatory activities. Due to its modulatory effect on inflammation, IL-6 is associated with cancer development [28]. In patients with untreated metastatic or castration-resistant prostate cancer (CRPC), the serum level of IL-6 is increased and also correlates negatively with tumour survival and response to chemotherapy. In vitro and in vivo studies showed that IL-6 promote prostate cancer cell proliferation and inhibit apoptosis through several cellular pathways, such as (1) the Janus tyrosine family kinase (JAK-signal transducer and activator of transcription (STAT) pathway, (2) the extracellular signal-regulated kinase 1 and 2 (ERK1/2)-mitogen activated protein kinase (MAPK) pathway, (and 3) the phosphoinositide 3-kinase (PI3-K) pathway [84]. Additionally, IL-6 is associated with aggressive prostate cancer phenotype and may be involved in the metastatic process through regulation of the epithelial–mesenchymal transition (EMT) and homing of cancer cells to the bone [84]. Similarly, Okamoto et al.’s [85] in vitro experiments pointed that IL-6 acts as a paracrine growth factor for androgen-sensitive and slow-growing prostate cancer cell lines (LNCaP) and as an autocrine growth factor for androgen-insensitive and fast-growing prostate cancer cell lines (DU145 and PC3).

### 5.6. Tumor Necrosis Factor α (TNF-α)

TNF-α is an adipokine of multidirectional action [86]. Previous studies showed that TNF-α presents of different actions in prostate cancer (pro- and anti-carcinogenic) [87]. TNF-α expression levels correlate with disease progression. The highest expression of TNF-α was observed in prostate cancer as compared to benign prostate hyperplasia [88]. Maolake et al. [89] results showed that TNF-α acts as a regulator of increased tumour cell migration through the upregulation of C-C chemokine receptor 7 (CCR7). Moreover, TNF-α in involved in the initiation of castrate-resistant prostate cancer by inducing hypersensitivity to androgen (in vitro LNCaP cells). Contrarily, anti-cancer activities include, among other activities, the inhibition of angiogenesis at high doses of TNF-α in in vitro and in vivo studies [90]. TNF-α is chronically produced at low levels within the tumour microenvironment [91]. Additionally, stimulates antitumour immunity by enhancing the generation and proliferation of cytotoxic T cells [91]. Additionally, Lee et al.’s [92] results showed that TNF-α induces apoptosis of androgen-sensitive, non-metastatic prostate cancer cell lines (LNCaP).

### 5.7. Vascular Endothelial Growth Factor (VEGF)

The physiological activity of VEGF consists of angiogenesis, development, wound healing, and haematopoiesis. VEGF presents pro-tumour properties. The angiogenesis and expansive vascularization are a critical process which determine the growth and progression of tumour. The most potent pro-angiogenic factor is VEGF-A. Previous studies showed that the inhibition of VEGF-dependent pathways may lead to regression of vascular network and inhibition of a tumour growth [93]. Duque et al. [94] study indicates that patients with metastatic prostate cancer have higher plasma VEGF levels than patients with localized disease or healthy controls. Wang et al.’s [95] results showed that the Neuropilin-2 (NRP2), which functions as a VEGF receptor on tumour cells, is an attractive target to activate antitumor immunity in prostate cancer because VEGF-NRP2 signalling sustains PD-L1 expression. Moreover, the blockade of the binding of VEGF to NRP2 using a mouse-specific anti-NRP2 monoclonal antibody led to necrosis and tumour regression in mouse prostate cancer models. Adipose-derived mesenchymal stem cells (ADSCs) play a critical role in vascularization. ADSCs secrete a wide range of cytokines and growth, angiogenic, and antiapoptotic factors. The angiogenic and antiapoptotic properties are exacerbated under hypoxemic conditions [96,97]. Systemic insulin, IGF-1 and other biomolecules (e.g., HIF-1, VEGF, etc.) may modify ADSCs homeostasis within PPAT, with further effects on prostate cancer biology.

### 5.8. Bone Morphogenetic Proteins (BMPs)

BMPs belongs to multifunctional molecules which acts as a modulators of cell differentiation, proliferation, survival, and motility. BMPs play important roles in the development and progression of prostate cancer, breast cancer and lung cancer. Additionally, BMPs are involved in cancer-related angiogenesis. It is believed that BMP can either directly regulate the functions of vascular endothelial cells or indirectly influence the angiogenesis via regulation of angiogenic factors, such as VEGF [98]. The higher expression of BMP-6 is closely related to higher-grade primary tumours and metastatic prostate cancer. Moreover, BMP-6 plays a role in the progression of castration-resistant prostate cancer [99,100]. Contrarily, Kobayashi et al.’s [101] results showed that BMP-7 plays a critical role in tumour dormancy and recurrence. The elevated level of extracellular BMP-7 suppresses the migration and invasion of tumour cells. BMP-7 counteracts physiological epithelial-to-mesenchymal transition. The expression of BMP-7 in prostate cancer tissue was strongly down-regulated compared with normal prostate tissue [102]. Additionally, the BMP7 administration inhibited the growth of cancer cells in bone in mouse model [100].

BMPs are secretory proteins which are involved in morphogenetic activities and cell differentiation throughout the body, including the development of adipose tissue and adipogenic differentiation. BMP-4 and BMP-7 are related to white adipogenesis and brown adipogenesis, respectively, but other BMPs (e.g., BMP-2, BMP-6, and BMP-8b) have been shown to also affect adipogenesis [103]. BMPs induce various effects by regulating multiple types of cells and orchestrating them to remodel adipose tissue for adaptation [104]. Adipocyte turnover is relevant to the morphology and function of adipose tissue as well as the physiological and pathological states of adipose tissues [105]. The low adipocyte generation rates are associated with adipose tissue overgrowth without an increased number of cells (hypertrophy), whereas high generation rates are associated with adipose hyperplasia. Hypertrophic adipocytes resulting from lipid overload induce a series of adverse effects, such as hypoxia, lipotoxicity, inflammation, and metabolic impairment [104,105]. Differentiation generates new adipocytes, and cell death clears old or abnormal adipocytes. The undoubtable impact of BMPs on adipocytes may also have an impact on the activity of PPAT, in consequence leading to secretome profile changes.

### 5.9. Mitochondrial Uncoupling Protein (UCP)

**Figure 3:**
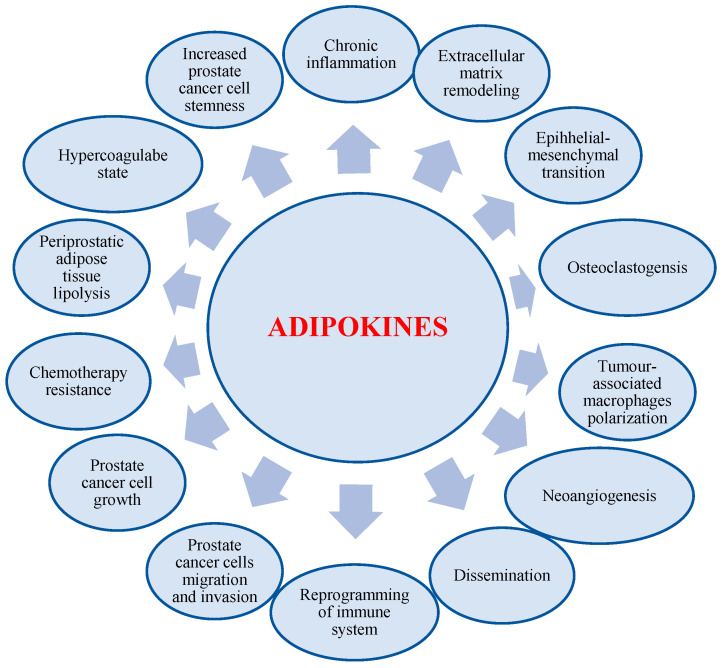
The multidirectional action of adipokines released by periprostatic adipose tissue (e.g., chemokines, cytokines, angiogenic factors, and other substances (e.g., leptin, adiponectin, IGF1, HIF-1α, etc.) in prostate cancer pathogenesis.

Uncoupling proteins (UCPs) 1–3 are mitochondrial anion carrier proteins, which play a crucial function in diminishing reactive oxygen species production [106]. Mitochondrial uncoupling protein 2 (UCP2) uncouples electron transport from ATP production. UCP-2 play an important role in obesity and diabetes. In prostate cancer tissue the levels of UCP-2 were significantly higher than that in the adjacent normal tissues [107]. Sadehgi et al. [108] results showed that the increased UCP-2 expression was correlated with decreased survival in prostate cancer. UCP-2 reprograms the immune state of the tumour microenvironment by altering its cytokine milieu in an interferon-regulatory-factor-5-dependent manner [109]. Tumour-associated macrophages (TAMs), due to modulation of tumour microenvironmental features, promote tumour progression and metastasis development [110]. Macrophages present an expression of UPC-2 [111]. Macrophages have a very interesting property that allows them to adapt to hypoxemic conditions in conjunction with tumour growth and local ischemia. Despite hypoxia, macrophages can adapt to these conditions, reprogramming their metabolism and being still activated and promoting further tumour growth [112] (Figure 3).

## 6. MicroRNAs

MicroRNAs (miRNAs) are single-stranded non-coding RNA molecules that play a regulatory role in gene expression and cancer cell signalling. The miR-628-5p (miR-628) was identified as a potential biomarker in serum samples from men with prostate cancer [113]. Srivastava et al.’s [114] results showed exposure to leptin, downregulated the expression of miR-628, and increased cell proliferation/migration in prostate cancer cells. miR-21 contributes to androgen-dependent and androgen-independent prostate cancer growth [115,116]. Dybos et al. [117] study showed that miR-148a-3p is upregulated in men with prostate cancer, and the miRNA is differentially expressed in patients with prostate cancer compared to healthy controls. miR-148a is an androgen-responsive microRNA that promotes LNCaP prostate cell (androgen-sensitive cells) [118]. He et al. [119] clinical study showed that serum miR-148a-3p level positively correlates while miR-485-5p level negatively correlates with prostate cancer’s progressing and postoperative recurrence. Considering the impact of diet and dairy on the risk of developing prostate cancer, it is worth mentioning that cow milk contains large amounts of microRNAs (miRNAs) [120]. miR-17-5p, miR-25, miR-27b, and miR-9-5p were significantly reduced in ultra-high-temperature-treatment milk (*p* < 0.05) but not significantly affected in pasteurized milk (except miR-27b) [121]. Zhang et al. [121] described that the bioactive miRNA in raw milk was lost after ultra-high-temperature treatment but not in pasteurized treatment. miR-17-5p has been reported to increase cell proliferation and the risk of metastasis in prostate cancer. Additionally, a high cancer cell expression of miR-17-5p was an independent negative prognostic factor in prostate cancer [122].

## 7. PPAT and Extracellular Matrix Remodelling

The two-way interaction between PPAT and prostate cancer is predisposed to the modification of the cancer microenvironment in favour of cancer development. Each component of PPAT, which consists of adipocytes, an extracellular matrix (ECM) and immune cells plays a role in this process. ECM due to specific architecture plays role in cell adhesion, as well as tissue regeneration and repair. Moreover, ECM acts in cell proliferation, migration, and apoptosis. Obesity leads to adipocytes hypertrophy as a consequence of excessive lipid accumulation. It provokes further changes, such as (1) local infiltration by immune cells, (2) an increased accumulation of collagens, elastin, and fibronectin in ECM (fibrosis), and (3) inflammation response. These processes seem to be strictly related to local hypoxia and insulin resistance [123]. Cancer development is predisposed to dysregulation of the ECM architecture and its function. The most common component of ECM is collagen, which provides the basic mechanical and functional properties of ECM. Developing cancer cells release several molecules such as collagenases and metalloproteinases leading to significant changes in the deposition or degradation of collagen and in a consequence the loss of ECM homeostasis. Previously, it was believed that collagen, a component of ECM, was only a passive barrier protecting cancer cells from developing cancer. However, it is now known that it plays an active role in its development and progression [124,125]. A dynamic interplay between the microenvironment and cancer cells is observed, leading to structural changes of the surrounding ECM during cancer cell proliferation. Increased secretion of fibronectin and different types of collagens (I, III, and IV) indicates that tumour progression demand a continuous interaction between the ECM and tumour cells [126]. Additionally, Paszek et al. [127] revealed that increased deposition of matrix proteins promotes tumour progression by interfering with cell-to-cell adhesion, cell polarity, and ultimately amplifying growth factor signalling. 

The tumour scaffold is based on collagen. ECM remodelling by collagen degradation and re-deposition affects tumour microenvironment, as well as promotes tumour infiltration, angiogenesis, invasion and migration [124]. Collagen changes in ECM release biomechanical signals, which are sensed by both tumour cells and stromal cells, trigger a cascade of biological events [124]. It is worth noting that a slight disturbance in tumour microenvironment homeostasis can have significant effects on the proliferation of cancer cells [93].

## 8. PPAT and Prostate Cancer Aggressiveness and Progression

In obesity, the normal balance of adipose tissue (including PPAT) secretory proteins is perturbed. This condition is associated with a greater risk of aggressive prostate cancer with increased local dissemination [24,128]. Su et al.’s [129] study establishes adipose stromal cells derived from white adipose tissue as the main source of CXCL12 driving prostate cancer aggressiveness by increasing the survival and proliferation of cancer cells. Through CXCR4-mediated signalling, the epithelial–mesenchymal transition (EMT) is also promoted, which intensifies the aggressiveness of prostate cancer. Another pathophysiological mechanism that enables the expansion of the prostate cancer tumour is the change in the structure of the extracellular matrix with the participation of PPAT. The adipocytes in PPAT through collagenases (CLG) and metalloproteinases (MMPs) leads to extracellular matrix remodelling, which, in conjunction with EMT, favours cancer cells migration out of the primary niche and their further dissemination [28]. Previous clinical studies showed that in comparison with normal weight patient, the obese individuals are at higher risk of prostate cancer progression after radical prostatectomy. Moreover, this cohort more likely develop metastatic prostate cancer and/or the cause of death will be prostate cancer [130]. These observations undoubtedly indicate that obesity is closely related to a more aggressive form of prostate cancer with a greater potential for clinical progression. Ribeiro et al.’s [130] study showed that the PPAT modulates extra-prostatic tumour cells’ microenvironment through increased MMPs activity and to promote prostate cancer cell survival and migration. Matrix metalloproteinases are associated with cancer-cell invasion and metastasis due to its proteolytic features [131]. MMP9 elicit epithelial-to-mesenchymal transition in tumour cells, leading to more aggressive phenotype of cancer [132]. The extra-capsular extension of cancer cells into the periprostatic area is a factor of early disease recurrence and worse prognosis in patient with prostate cancer [133]. Sacca et al. [134] study showed that PPAT releases pro-MMP-9 which induces the invasive capacity of androgen dependent prostate cancer cell lines (LNCaP cells). Therefore, it is postulated that PPAT via MMP-dependent pathways may modulate prostate cancer progression even from the early stages of the disease. Previous studies showed that obesity is more related to the aggressiveness of the tumours and not directly related to the risk of development of prostate cancer. Moreover, prostate cancer progression depends on tumour activity as well as the active function of the microenvironment [135,136]. In obese patients with prostate cancer, the risk of cancer progression is more potent due to chronic inflammation, which is strictly related to increased secretion of pro-inflammatory adipokines and overproduction of reactive oxygen species. PPAT, which is a part of surrounding microenvironment for prostate cancer, participate in the bi-directional interplay with cancer cells via paracrine route. Moreover, the molecules excreted by cancer cells, stromal cells, adipocyte, and adipose stem cells into the tumour microenvironment play a critical function in cancer progression. Sacca et al.’s [137] study provides that in prostate cancer the metabolic dysfunction of PPAT occur. The prostate cancer microenvironment is altered by adipokines and fatty acids (FAs) secreted by PPAT. In locally advanced prostate cancer (T3 stage) the secretome of PPAT is enriched in several pathways related to energy production, indicating a greater energy requirement by the tumour, when compared to the organ-confined prostate cancer (T2 stage) [137]. Moreover, T3 stage prostate cancer presents enrichment in pathways related to hormone response, polyamine synthesis, and control of protein synthesis through amino acid, RNA, and nucleotide metabolism [137]. Balaban et al. [138] results revealed interesting feature of prostate cancer cells. These cells use extracellular FAs as both fuel for oxidation and the primary substrates for complex lipid synthesis such as triacylglycerols, and that high extracellular lipid availability further enhances FA flux in these cells. Iordanescu et al. [139] clinical study showed the linear association between periprostatic adipose tissue fatty acid composition and extracapsular extension of prostate cancer and is marked by elevated monounsaturated and reduced saturated fatty acids in the PPAT relative to subcutaneous adipose tissue. The pathological stage of prostate cancer determines the different FA compositions of PPAT, leading to alteration in proper lipid metabolism [137]. It is postulated that PPAT remains an important reservoir of FAs for prostate cancer cells. In accordance with Yue at al.’s [140] observations, the expression of several genes related to lipid metabolism (e.g., CD36) was decreased when PPAT explants from patients with prostate cancer who underwent radical prostatectomy were co-cultured with prostate cancer cell lines. This strictly indicates a progressive decline in PPAT lipid production and utilization. Additionally, it is worth noting that increased lipid absorption and more potent lipid accumulation in prostate cancer cells, which secondarily leads to an increased number of intracellular lipid droplets (esterified cholesterol), was associated with more aggressive potential of prostate cancer (high-grade prostate cancer and metastases) [140]. In vitro study in prostate cancer cell lines showed that the loss of tumour suppressor PTEN and subsequent activation of the phosphatidylinositol 3-kinase (PI3K)/AKT pathway led to extensive cholesteryl ester accumulation. Contrarily, in a mouse animal model, the inhibition of cholesteryl ester storage significantly reduced cancer proliferation, impaired cancer invasion capability, and suppressed tumour growth [140]. FA uptake was increased in human prostate cancer via the CD36 fatty acid transporter. The modulation of fatty acid transporter CD36 activity ameliorates free FA uptake and consequently diminishes the prostate cancer progression [141,142]. 

Several pathomechanisms of prostate cancer progression (described as tumour growth, local invasion, and bone metastasis development) were defined as follows: (1) neovascularisation, (2) immunosuppression and macrophage phenotype modification, (3) dissemination growth, and (4) osteoclastogensis. PPAT secretes many molecules, which regulate all of the above mentioned processes. The neovascularisation is mediated via several adipokines, e.g., VEGF, basic-FGF and IL-8). Immunosuppression is regulated by IL-10 and TGF-β. Yunna et al. [143] described that macrophages may change their phenotypes (M1 and M2 populations) due to variety of factors. M1-typed macrophages are mostly involved in the pro-inflammatory response, contrary to the M2 type, which presents anti-inflammatory features. The polarisation of the tumour-associated macrophage (TAM) population is affected by IL-4 and IL-13. In cases of more advanced stages of prostate cancer, dissemination growth, and osteoclastogenesis in critical in-bone metastasis development. The former process is regulated by IL-6, IL-8, TNF-α and TGF-β. On the other hand, the latter process is influenced by parathyroid hormone-related protein (PTHrP), IL-1, IL-6, TNF-α and TGF-β.

It is worth nothing that a large group of patients diagnosed with prostate cancer were previously treated with 5α-reductase inhibitors, which may also affect the biology of prostate cancer. This appears to be consistent with Taussky et al.’s [144] results, which showed that 5α-reductase inhibitor treatment for at least 12 months reduces PPAT volume. Moreover, it points that 5α-reductase inhibitors may affect the prostate cancer biology through modulation of PPAT activity.

Prostate-specific antigen (PSA) expression level in prostate cancer cells is one of the strongest prognostic features in this tumour entity [145]. PSA is an insulin-like growth factor binding protein-3 protease. Thus, PSA may modulate IGF-dependent pathways function by altering IGF-IGFBP-3 [146]. There are no clear data on the occurrence of PSA expression on the adipocytes that form PPAT. However, prostate cancer cells present a positive expression of PSA [145]. Additionally, such expression may lead to crosstalk between prostate cancer cells and PPAT by modulatory function on PPAT secretome through the IGF-1 dependent pathway.

The PPAT contributes through paracrine mechanisms to prostate cancer progression and affects its aggressiveness [147]. Detection of PPAT thickness above 3 mm was observed to be an independent risk factor for upstage in prostate cancer patients [148]. Jiang et al. study [149] showed that the PPAT thickness (PPATT)/subcutaneous adipose tissue (SAT) thickness (SATT) ratio is an independent risk factor for biochemical recurrence after radical prostatectomy. This indicates that patients with thicker PPATT and thinner SATT have a higher probability of biochemical recurrence. This clinical study strictly highlights the role of PPATT and overall fat distribution in predicting biochemical recurrence and overall survival in patients with prostate cancer. Moreover, patients with higher PPATT/SATT ratio had relatively higher Gleason scores (total score ≥ 9, 81.6% vs. 68.3%). The PPATT/SATT ratio > 0.22 is an independent risk factor for biochemical recurrence after radical prostatectomy. Additionally, van Roermund et al. [150] showed a significant association between periprostatic fat density and risk of having a high-risk disease in T1-3N0M0 prostate cancer patient qualified to radiotherapy. Similarly, Shao et al. [73] study showed that PPAT was significantly associated with extracapsular prostate cancer extension. Furthermore, in vitro experiments revealed that PPAT could inhibit prostate cancer cell proliferation by secreting factors that activated immune responses and could thereby promote cancer cell apoptosis. This mechanism may act as a first line of defence in the early stages of prostate cancer [73].

## 9. Thrombotic Cascade Activation via PPAT and Prostate Cancer Progression 

In obesity, the insulin resistance and excessive caloric intake lead to adipose tissue expansion and adipocytes hypertrophy, and in consequence reduce adipose tissue oxygenation. Localised hypoxia increases the adipocyte cell death and stimulate the local adipocyte tissue inflammation which is corelated with extensive inflammatory cytokines and adipokines release. Previous studies showed that in obesity-related disorders (e.g., cardio-vascular diseases, diabetes, and cancer), a hypercoagulable state seems to be a contributor in their pathogenesis. In obesity, the inflammation within adipocyte tissue provides increased expression and activation of factor X (FXa) and thrombin as well as different isoforms of the protease-activated receptors (PARs), leading to a hypercoagulable state [151]. It is postulated that exacerbated inflammation process in PPAT predispose to prostate cancer phenotype progression including through thrombotic cascade activation. Ebrahimi et al. [152] described that inflammation induced via thrombin-dependent pathway facilitate tumour cells proliferation, angiogenesis, invasion, and metastasis. These processes are caused through increased expression of cytokines, adhesion molecules, angiogenic factors, and matrix-degrading proteases. The activation of coagulation cascade is boosted via tissue factor (TF) and factor VII. Moreover, adipocyte tissue is a major source of TF. The level of TF is increased in chronic inflammation [153]. Prostate (prostate epithelium and the stroma) is a rich reservoir of thrombin. Thrombin procoagulant activity is associated with more advanced disease and a worse prognosis in patients with prostate cancer [154]. It is worth noting that cellular effects of thrombin are mediated by PARs [155]. Additionally, improper expression of PARs provides increased angiogenesis, tumour growth, and metastasis of various cancers. Kaushal et al. [156] resulted revealed that PAR-1 expression is increased in advanced-stage of prostate cancer and is localized to endothelial cells in the vascular network of prostate tumour areas. Moreover, Black et al. [157] showed that PAR mRNA expression in prostate specimens after radical prostatectomy was higher in cancer, and protein expression was increased in PAR-1 (45%), PAR-2 (42%), and PAR-4 (68%) compared to normal prostate glands. Moreover, increased expression of PAR-1 in periglandular stroma of the prostate was associated with higher rates of biochemical recurrence in prostate cancer. 

Additionally, the thrombin-dependent pathway affects the macrophage population. Thrombin promoted the differentiation of macrophages into an M1-like phenotype polarization with an increased production of proinflammatory cytokines. The cellular actions of thrombin on macrophages were mediated in part by PAR-1 [158]. 

Additionally, the increased lipolysis in PPAT, evoked by thrombin, release free FAs, which participate in the development of oxidative stress in the prostatic tissue and exacerbation of inflammatory process [151]. Prostate cancer cells accumulate the released free FAs, which enhance the invasive potency of cancer cells. This triggers a further cascade consisting of the induction of an isoform of NADPH oxidase (NOX5) and intracellular ROS production. In the next stage, the HIF-1α/MMP-14 signalling pathway is launched via intracellular ROS [151,159]. In obesity, the tumour-surrounding adipocytes are more prone to activating the HIF-1α/MMP-14 signalling pathway. Additionally, the expression of NOX5 and MMP-14 is upregulated in areas of the tumour that are in the proximity of the PPAT [159].

## 10. Other PPAT Molecules and Prostate Cancer Pathogenesis 

In addition to the previously discussed molecules that have an established role in the development of prostate cancer and obesity, there are many molecules in the literature that may influence the pathogenesis of prostate cancer and obesity (e.g., visfatin, omentin, resistin, LCN2, FABP4, osteopontin, etc.) [151]. The importance of these molecules in prostate cancer is outlined below.

### 10.1. Visfatin

Macrophages which infiltrate adipocyte tissue are characterized by increases level of visfatin. Previous studies showed that upregulation of visfatin in malignancies (e.g., in prostate cancer). Marked visfatin expression was detected in human locally advanced prostate cancer tissues (with extraprostatic extension) compared to minimal expression of organ-confined diseases. Visfatin plasma concentration was elevated in patients with prostate cancer. Additionally, the greater level of visfatin was observed in cases of prostate cancer with extraprostatic extension compared to organ-confined disease [160]. Moreover, the results of in vitro cell culture and in vivo preclinical mouse model studies performed by Sun et al. [160] confirmed visfatin-mediated enhancement of prostate cancer invasiveness into muscle tissues. The blockade of visfatin by polyclonal antibodies leads to the suppression of prostate cancer invasiveness.

### 10.2. Omentin

Omentin (intelectin-1) was identified at higher plasma concentration in patients with different malignancies, as well as is collated with insulin resistance. The visceral adipose tissue synthetizes intelectin-1. Uyeturk et al. [161] study showed elevated level of circulating omentin in patients with prostate cancer compared to patients with benign prostate hyperplasia. The role of omentin in prostate cancer still remains unclear. 

### 10.3. Resistin

Resistin (ADSF–adipose-tissue-specific secretory factor) peptide hormone derived from adipose tissue which characterize proinflammatory properties. This adipokine links obesity and diabetes by prompting insulin resistance. Moreover, ADSF plays a role in inflammation, proliferation, angiogenesis, and metastasis and also affects the cancer cells’ metabolism [151,162]. Kim et al. [163] revealed that human prostate cancer cell lines PC-3 and DU-145 express human resistin mRNA. Additionally, in comparison to benign prostate hyperplasia, the increased level of resistin was observed in high-grade prostate cancer tissue. Several potential ADSF-dependent mechanisms affecting cancer biology (by promotion of cancer progression and/or increasing its aggressiveness) have been proposed, as follow: (1) invasion and metastasis, (2) epithelial-to-mesenchymal transition (EMT) and stemness, (3) angiogenesis, and (4) chemoresistance [164]. 

### 10.4. Lipocalin-2 (LCN2)

LCN2 is characterized by multidirectional action. LCN2 participates in cell membrane transport, modulates immune responses, and promotes epithelial cell differentiation. Although LNC2 is expressed at low levels in most human tissues, it is abundant in aggressive subtypes of several cancer (e.g., breast, colon, pancreas, thyroid, etc.). LCN2 has been associated with increased cell proliferation, angiogenesis, cell invasion, and metastasis [165]. Moreover, LCN2 is elevated in obesity [151]. Ulusoy et al.’s [166] results indicate that high LCN2 expression was strictly correlated with higher prostate cancer grade (Gleason score). LCN2 knockdown in PC3 and DU145 prostate cancer cells decreased cell proliferation, colony formation, cell cycle arrest, migration, and invasion [167]. Additionally, Ding et al.’s [168] results showed that LCN2 could facilitate cell proliferation of castration-resistant prostate cancer (CRPC) via androgen receptor transcriptional activity. Moreover, LCN2 promotes cell migration and invasion of prostate cancer by inducing epithelial-to-mesenchymal transition via the extracellular signal-regulated kinase (ERK)/SLUG pathway. The positive correlation between higher concentration of LCN2 and prostate cancer invasiveness in in vivo and in vitro studies [169]. SLUG is a zinc-finger transcription factor that plays a role in migration and invasion of tumour cells. SLUG promotes prostate cancer cell migration and invasion via CXCR4/CXCL12 axis [170].

### 10.5. Fatty Acid Binding Protein 4 (FABP4)

FABP4, a member of the cytoplasmic fatty acid binding protein multigene family is secreted by adipocytes. The expression of FABP4 is increased in metabolic syndrome and in obesity [171]. Adipocytes release FABP4. Uehara et al. [171] showed that FABP4 treatment promoted serum-induced prostate cancer cell invasion in in vitro model. Moreover, in prostate cancer animal model the blockade of FABP4 diminished the tumour growth and metastasis, partly by inducing prostatic epithelial cell DNA damage and apoptosis. Elevated expression of FABP4 and secretion of FABP4 by prostate cancer cells trigger the invasiveness of the prostate cancer by stimulation of matrix metalloproteinases (MMPs) via phosphatidylinositol 3-kinase (PI3K) and mitogen-activated protein kinase (MAPK) signalling pathways [172]. Additionally, FABP4 facilitates prostate cancer aggressiveness through increased secretion of IL-6 and IL-8 [173].

### 10.6. Osteopontin (OPN)

OPN is an inflammatory cytokine, the expression of which is strongly upregulated in adipose tissue and liver upon obesity [173]. Prostate-cancer-afflicted individuals had higher plasma levels of OPN compared to patients with benign prostate hyperplasia [174]. Moreover, poor prognosis in prostate cancer was strictly associated with higher concentration of OPN [175]. OPN presents pro-tumorigenic effect through the activation of several signalling pathways after binding to integrin αvβ3. Moreover, OPN upregulates CD44 and MMP-9 expression, providing a more potent ability for cancer cell invasion and metastasis development [176,177]. Khodavirdi et al.’s [178] results strictly indicated that OPN contributes to several steps in the process of prostate carcinogenesis and metastasis. Prostate cancer tumours with OPN overexpression seem to present an increased proliferative and invasive feature. It is postulated that prostate cancer progression is also mediated through OPNc, which activates androgen receptor signalling [179]. Prostate cancer cell culture (PC-3) with OPNc overexpression presents significantly induced endothelial cell adhesion, proliferation and migration [180]. As expected, decreased expression of OPN in PC-3 cell culture induced apoptosis of cell culture, leading to suppression of malignant potency of the cell colony [181]. 

## 11. PPAT, Chronic Hypoxemic State and Prostate Cancer 

In obesity, the expression of HIF-1α in adipose tissue is increased. The HIF-1α activity is stimulated by several factors as follows: (1) adipogenesis, (2) insulin, and (3) hypoxia. Moreover, HIF-1α is a major transcriptional activator for the VEGF gene, but it is not sufficient for activation of VEGF gene expression in adipose tissue [182]. Additionally, García-Fuentes et al. [183] showed that morbidly obese subjects have a higher level of visceral adipose tissue HIF-1α. In comparison to other adipose tissue, PPAT has a sparse vascular network responsible for a chronic hypoxic state, leading to inflammation as well as fibrosis independent of obesity. Roumiguié et al. [184] study revealed an increase in expression of the oxygen-labile HIF-2α, but not of HIF-1α, in PPAT, indicating an adaptive response of this tissue to hypoxia. The higher level of VEGF secretion by PPAT when compared with adomino-pelvic adipose tissue may promote angiogenesis. Additionally, due to chronic hypoxia the PPAT exhibits hallmarks of chronic inflammation. Hypoxia-inducible factors (HIFs) play a pivotal role in cancer cell adaptation to hypoxic environments, contributing to treatment resistance. Peng et al.’s [185] in vitro studies showed that the marine-derived HIF-1α inhibitor reduces prostate cancer cell proliferation by targeting HIF-1 target genes.

## 12. PPAT, Diet and Prostate Cancer

PPAT is believed to be a so-called driving force for prostate cancer progression [186]. PPAT releases a wide range of molecules, which promotes tumour growth and the migration of cancer cells. However, some molecules released from PPAT, such as adiponectin and the n-6 or n-3 polyunsaturated fatty acids, have been shown to have anti-tumour properties. This fact points that the interplay between PPAT and prostate cancer biology depend on the balance between the pro- and anti-tumour components of PPAT [187]. Additionally, obesity and dietary factors seem to be involved in the risk and aggressiveness of prostate cancer through the modulatory impact on the interactions between PPAT and cancer cells and their consequences on the growth and the metastatic potential of prostate cancer.

The anti-inflammatory properties of vitamin D seem to be important in cancer prevention [188]. However, the results are conflicting and inconsistent [189]. Current data show an active role of vitamin D and its metabolites in physiological adipocyte and adipose tissue processes in adulthood. Vitamin D seems to affect the adipogenesis, energy metabolism, and inflammation process [190]. Insulin-like growth factor (IGF)-1 and its binding proteins are important in cancer growth, especially in prostate cancer [191]. The meta-analysis performed by Kord-Varkaneh et al. [192] indicates a non-significant increase in IGF-1 following vitamin D supplementation. Additionally, vitamin D dosages of <1000 IU/day and intervention durations of <12 weeks significantly raised IGF-1 levels. Additionally, Guzey et al. [193], in an animal model study, showed that vitamin D receptor deficiency in mice with prostate induces fat necrosis and individual cell apoptosis in PPAT, which regulates prostate cancer signalling pathways and affects prostate cancer progression.

Previous studies show that high-fat dairy, particularly whole milk, in healthy men may increase risk of aggressive prostate cancer. The potential impacts of a dairy diet on the risk of prostate cancer development are as follow: (1) high calcium intake decreasing vitamin D levels, (2) increased level of IGF-1 levels, (3) fluctuating phosphorus levels modifying vitamin D3 concentrations, and (4) elevated saturated fat intake modulating the immune response and inflammation [194]. Moreover, whole milk consumption after prostate cancer diagnosis was associated with increased risk of recurrence, particularly among very overweight or obese men [194]. Daily milk consumption in adolescence, but not in midlife or currently, was associated with a 3.2-fold risk of advanced prostate cancer [195]. Additionally, Song et al.’s study [196] showed that higher intake of skim/low-fat milk was associated with a greater risk of nonaggressive prostate cancer. Most importantly, only whole milk was consistently associated with higher incidence of fatal prostate cancer in the entire cohort and higher prostate cancer-specific mortality among cases. Similarly, men with higher intake of dairy foods, but not nondairy calcium, had a higher risk of prostate cancer compared with men having lower intakes [197]. Zhao et al.’s [197] meta-analysis showed an increased risk of prostate cancer with high intakes of total dairy products, milk, cheese, and butter. Dietary factors, especially cow milk consumption and dairy products, which raise systemic IGF-1 levels and donate abundant amounts of essential branched-chain amino acids of the leucine prototype may affect prostate cancer development. Increased insulin/IGF-1 signalling may promote tumour growth directly through insulin receptors or regulation of IGF and their binding proteins (IGFBP), which are involved in cell survival and proliferation [198]. Prostate cancer is dependent on androgen receptor signalling and aberrations of the PI3K-Akt-mTORC1 pathway mediating excessive and sustained growth signalling. The nutrient-sensitive kinase mTORC1 is upregulated in nearly 100% of advanced human prostate cancers, and this kinase is activated by leucine, insulin, IGF-1, and glucose. Oncogenic mTORC1 signalling activates key subsets of mRNAs that cooperate in distinct steps of prostate cancer initiation, cell growth, and progression [199]. Only milk proteins in comparison to meat and fish have the unique ability to preferentially increase both the insulin/IGF-1 and the leucine signalling pathways necessary for maximal mTORC1 activation [199]. mTORC1 activation is critically dependent on the availability of sufficient amounts of amino acids, especially of the branched-chain essential amino acid (BCAA) leucine [200]. Castration-resistant prostate cancer cells are addicted to leucine availability [201,202]. L-type amino acid transporters (LATs) uptake neutral amino acids, including L-leucine, into cells, stimulating mTORC1. LAT1 and LAT3 are overexpressed at different stages of prostate cancer, and they are responsible for increasing nutrients and stimulating cell growth. LAT inhibition suppressed M-phase cell cycle genes regulated by E2F family transcription factors including critical castration-resistant prostate cancer regulatory genes [203].

## 13. PPAT and Prostate Cancer Treatment (Radiotherapy, Androgen Deprivation Therapy, Chemotherapy)

Adipose tissue in the tumour microenvironment impacts the efficacy of chemotherapeutic agents and androgen deprivation treatments [204,205]. Androgen deprivation treatment (ADT) induces oxidative stress and inflammation in adipose tissue. This causes an increase in adipose tissue, cholesterol, oxidative stress, and inflammation, leading to tumour progression. Obesity-related proteins—thioesterase superfamily member 6 (THEM6) and yes-associated protein 1 (YAP1)—are associated with ADT resistance in prostate cancer by increasing cellular lipid content and activating the UPR [206,207]. THEM6, which is highly expressed in adipose tissue where it regulates intracellular fatty acid homeostasis, is upregulated in ADT-resistant prostate cancers, and activates the unfolded protein response (UPR) [208]. ADT affect the homeostasis of adipocyte tissue. Previous studies showed that ADT is a risk factor of insulin resistance and affect the adiponectin level [200,201]. Lam et al. [209] revealed that, in patients receiving ADT for 6 months, the PPAT presents a significant trend of pro-inflammatory, obesity-like adipose tissue. Obesity is a well-established risk factor for prostate cancer. Thus, ADT could potentially cause adverse effects on adipose tissue, linking it to tumour progression [206]. In case of radiotherapy, the irradiation to adipose tissue decreases lipogenic gene expression and increases reactive oxygen species and lipolysis. Both reactive oxygen species and lipolysis lead to the release of unsaturated fatty acids from the adipocytes, which cancer cells use to activate pro-cancer pathways. Additionally, radiation activates fat mass and obesity-associated (FTO) protein, which demethylates mRNA to promote radio-resistance [206]. Chemotherapy is often lipophilic and, therefore, easily sequestered and metabolized by adipocytes containing the aldo-keto reductase (AKR) protein, reducing the availability of the drug for prostate tumours [206,207]. Chemotherapy induces dysregulated cytokine secretion from adipose tissue, such as an increased release of IGF-1, causing the upregulation of TUBB2B, which is associated with chemo-resistance. PPAT secretes insulin-like growth factor-1 (IGF-1) leading to increased prostate cancer cell expression of TUBB2B, β-tubulin isoform 2B. TUBB2B is upregulated in metastatic prostate cancer and promotes docetaxel resistance by preventing drug-induced microtubule stabilization [206,210,211,212].

## 14. Exosome- and mTORC-Dependent Pathways and Prostate Cancer

Exosomes are extracellular vehicles (EVs) of endocytic and secretory exocytic origin that play a crucial role in cell-to-cell communication. Exosomes transport a wide range of cellular components (e.g., miRs, mRNAs, etc.) and are involved in different cancer development (e.g., breast, prostate, ovarian etc.) [213]. Primary human adipocytes shed EVs, specifically exosomes, that induced the expression of genes associated with epithelial-to-mesenchymal transition (EMT) and cancer-stem-like cell (CSC) traits in cocultured breast cancer cell lines [214,215]. Wang et al.’s [215] results showed that a mechanism of exosome-mediated cross-talk between metabolically abnormal adipocytes and breast cancer cells may promote tumour aggressiveness in patients with diabetes type 2. Exosomes regulate angiogenesis, immune suppression, metastasis, and drug and therapeutic resistance in prostate cancer [216]. Additionally, exosome release is stimulated by pH changes in prostate cancer microenvironment [216]. PPAT interplays with the tumour microenvironment via exosomes EVs. Sanchez-Martin et al.’s [217] study showed that the retinoic-acid-receptor-related orphan receptor alpha (RORA) gene was regulated by PPAT-EVs-derived miRNAs. The RORA gene has a role in prostate cancer cells proliferation and inflammation.

The mammalian target of rapamycin (mTOR) kinase is a master regulator of protein synthesis that couples nutrient sensing to cell growth and cancer. Several genes responsible for cancer cell metabolism, proliferation, and invasion regulated by mTOR signalling have been described [218]. Moreover, the complete mTOR inhibition by ATP site blockade reprograms this gene expression prevents prostate cancer invasion and metastasis [218]. Additionally, mTORC1 regulates polyamine dynamics, which are also important in carcinogenesis. In prostate cancer, alterations in the production of decarboxylated S-adenosylmethionine (dcSAM) and polyamine synthesis were observed. S-adenosylmethionine decarboxylase 1 (AMD1) was upregulated in prostate cancer specimens with activated mTORC1 [219]. Everolimus (the mTORC1 inhibitor) exhibited a predominant decrease in AMD1 immunoreactivity that was associated to a decrease in proliferation, in line with the requirement of dcSAM production for oncogenicity [220]. It is worth noting that in cases of prostate cancer, the activation of the phosphatidylinositol-3-kinase (PI3K), protein kinase B (PKB/AKT), and mTOR-dependent pathways facilitates tumour formation, disease progression, and therapeutic resistance. The complex crosstalk between the PI3K-AKT-mTOR pathway and multiple interacting cell signalling cascades can further promote prostate cancer progression and influence the sensitivity of prostate cancer cells to PI3K-AKT-mTOR-targeted therapies [221]. Regulation of the mTORC1 signalling pathway is performed by growth factors (through the insulin/IGF-1 pathway), energy (glucose level, AMP to ATP ratio changes, etc.) and oxygen levels [222]. In mouse models of prostate cancer, the signalling adaptor p62/Sqstm1 is selectively inactivated in adipocytes. p62 loss in adipocytes results in increased osteopontin secretion, which mediates tumour fatty acid oxidation and invasion, leading to aggressive metastatic prostate cancer in vivo. Furthermore, p62 deficiency triggers in adipocytes a general shutdown of energy-utilizing pathways through mTORC1 inhibition, which supports nutrient availability for cancer cells [223].

## 15. Tumour Microenvironment and Prostate Cancer

Several types of stromal cell support a tumorigenic primary niche. The immune system makes an attempt to eliminate tumour cells. Specific antigens of tumour cells play a role in this process and are recognized by cytotoxic immune cells, leading to their destruction. In the tumour microenvironment, macrophages and fibroblasts contribute to a growth-suppressive state; however, these cells may later become educated by the tumour to acquire pro-tumorigenic functions. On the other hand, tumour-associated macrophages (TAMs) support the primary tumour including growth, angiogenesis and invasion by secreting a wide range of pro-tumorigenic agents (e.g., proteases, cytokines and growth factors, etc.). Due to cytokine release during tumorigenesis the immune-suppressor cells are mobilized. Additionally, tumour cells release chemokines, which activate cancer-associated fibroblasts (CAFs). CAFs secrete extracellular matrix proteins and basement membrane components, as well as regulate differentiation, modulate immune responses, and contribute to deregulated homeostasis. Moreover, during the tumour growth the neo-angiogenesis is stimulated by VEGF released from CAFs. It is worth mentioning that parallelly to cellular alterations, the extracellular properties change (e.g., hypoxia, extracellular matrix components change) [224].

## 16. Conclusions

Current data strictly highlight the fundamental role of periprostatic adipose tissue (PPAT) and its active function in prostate cancer development and progression. The complexity of PPAT-related adipokines in prostate cancer pathogenesis points PPAT as a potential therapeutic target to modulate the interaction between the prostate cancer cells and microenvironment surrounding prostate gland. Considering the multipotential activity of PPAT and impact of prostate cancer biology, PPAT seems to become an interesting and promising avenue with potential therapeutic benefits in patients with prostate cancer.

## 17. Future Research Questions

The current epidemiological prognosis for the degree of obesity in the human population until 2050 is not optimistic [225]. This may have a huge impact on the incidence of prostate cancer. BMI > 25 kg/m^2^ is associated with an increased risk of prostate cancer and mortality [226]. Recently, there has been an intensive increase in the use of novel drugs in the treatment of obesity, such a liraglutide (glucagon-like peptide-1 (GLP-1) agonist) and tirzepatide (a glucose-dependent insulinotropic polypeptide (GIP) and GLP-1 receptors agonist). Taking into account the fact that there is a close relationship between obesity, PPAT, and prostate cancer, a new field is opened for research aimed at explaining GIP as well as GLP-1 agonist and what effect the novel agents used in the treatment of obesity have on the biology of prostate cancer.

## List of Abbreviations

ADSCs	adipose-derived mesenchymal stem cells
ADSF	adipose-tissue-specific secretory factor
AKR	aldo-keto reductase
AMD1	S-adenosylmethionine decarboxylase 1
BAT	brown adipose tissue
BCAA	branched-chain essential amino acid
BMI	body mass index
BMPs	bone morphogenetic proteins
CAFs	cancer-associated fibroblasts
CLG	colagenase
CRPC	castration-resistant prostate cancer
CSC	cancer-stem-like cell
CXCR4	chemokine receptor CXC type 4
dcSAM	decarboxylated S-adenosylmethionine
ECM	extracellular matrix
EMT	epithelial–mesenchymal transition
ERK1/2	extracellular signal-regulated kinase 1 and 2
EVs	extracellular vehicles
FABP	fatty acid binding protein
GIP	glucose-dependent insulinotropic polypeptide
GLP-1	glucagon-like peptide-1
HIF	hypoxia-inducible factor
IFG-1	insulin-like growth factor 1
IGFBP-1	IGF-binding protein 1
IL-1	interleukin-1
IL-6	interleukin-6
JAK	the Janus tyrosine family kinase
LATs	L-type amino acid transporters
LCN	lipocalin
MAPK	mitogen activated protein kinase
MMPs	metalloproteinases
mTOR	mammalian target of rapamycin
NK	natural killer
OPN	osteopontin
PPAT	periprostatic adipose tissue
PPATT	periprostatic adipose tissue thickness
PAR	protease-activated receptors
PI3K	phosphatidylinositol-3-kinase
PSA	prostate-specific antigen
PTEN	tumour suppressor phosphatase and tensin homologue
PTHrP	parathyroid hormone-related protein
P90RSK	90 kDa ribosomal S6 kinases
RORA	retinoic acid-receptor-related orphan receptor alpha
ROS	reactive oxygen species
SATT	subcutaneous adipose tissue thickness
STAT	signal transducer and activator of transcription
TAMs	tumour-associated macrophages
TF	tissue factor
THEM6	thioesterase superfamily member 6
TNF-α	tumour necrosis factor
TUBB2B	tubulin beta 2B class IIb
UCP	mitochondrial uncoupling protein
WAT	white adipose tissue
VEGF	vascular endothelial growth factor
